# Amyloid β deposition and glucose metabolism on the long-term progression of preclinical Alzheimer's disease

**DOI:** 10.4155/fsoa-2018-0069

**Published:** 2019-02-19

**Authors:** Shizuo Hatashita, Daichi Wakebe

**Affiliations:** 1 Department of Neurology, Shonan-Atsugi Hospital, Atsugi 243-0033, Japan; 2 Department of Radiology, Shonan-Atsugi Hospital, Atsugi 243-0033, Japan

**Keywords:** Alzheimer's disease, amyloid β deposition, glucose metabolism, preclinical AD

## Abstract

**Aim:**

Longitudinal changes in beta amyloid (Aβ) deposition and glucose metabolism over a long-term progression of preclinical Alzheimer's disease (AD) were evaluated.

**Methods:**

22 preclinical AD subjects with amyloid-positive scans underwent [^11^C]-labeled Pittsburgh Compound-B (PIB) positron emission tomography (PET) and [^18^F]-fluorodeoxyglucose (FDG) PET imaging over 6.0 ± 1.8 years. A quantitative analysis of [^11^C]-PIB and [^18^F]-FDG was used with a standardized uptake value ratio (SUVR) in the same regions.

**Results:**

In preclinical AD subjects, the cortical PIB SUVR was higher at baseline and increased at follow-up. 12 of the preclinical AD subjects progressed to mild cognitive impairment, six of whom had reduced glucose metabolism. The annual change in PIB SUVR was not related to that in FDG SUVR.

**Conclusion:**

Increases in Aβ deposition lead to the progression to mild cognitive impairment, but decreases in glucose metabolism do not contribute to progression.

## Lay abstract

Longitudinal changes in Aβ deposition and glucose metabolism were clarified over a long-term progression of preclinical AD using [^11^C]-PIB PET and [^18^F]-FDG PET imaging. 73% of the preclinical AD subjects progressed to mild cognitive impairment during 6 years of a long follow-up period. The increases in Aβ deposition lead to the progression to mild cognitive impairment but decreases in glucose metabolism do not contribute to progression.

The National Institute on Aging Alzheimer's Association (NIA-AA) workgroup has proposed diagnostic criteria for the spectrum of Alzheimer's disease (AD) supported by distinctive and reliable biomarkers of AD [1]. In cognitively normal (CN) individuals who have evidence of beta amyloid (Aβ) accumulation, the operational framework for the preclinical phase of AD has been demonstrated in an asymptomatic or latent form of the disease as preclinical AD [2]. The preclinical stage of AD has a time lag of a decade or more between the beginning of the AD pathophysiological process and the onset of evident clinical impairment. The long preclinical phase of AD allows potential intervention with disease-modifying therapy that treats the early pathological process to prevent subsequent neurodegeneration and eventual cognitive decline. It is important for the pathophysiological process in the preclinical stage of AD to be evaluated with molecular positron emission tomography (PET) imaging of Aβ accumulation and neurodegeneration.

Amyloid PET imaging with a tracer of [^11^C]-labeled Pittsburgh Compound-B (PIB), which has high affinity for fibrillar Aβ, is a reliable biomarker of Aβ accumulation [3]. We previously reported that a diagnostic framework with Aβ deposition by [^11^C]-PIB PET at different clinical stages of AD enables an earlier and more specific AD diagnostic process [4]. Furthermore, in the patients with mild cognitive impairment (MCI) due to AD defined by [11C]-PIB PET, our recent work has demonstrated that a substantial increase in brain Aβ deposition has an effect on cognitive decline and disease progression [5]. In addition, if CN individuals are identified as preclinical AD with a positive Aβ PET scan, they may be at risk for progression to MCI or AD dementia over a long term.

Fluorodeoxyglucose (FDG) PET imaging for cerebral glucose metabolism, which is one of the biomarkers for neurodegeneration, provides evidence of cognitive function and progression along the spectrum of AD. FDG PET has revealed a decrease in cerebral glucose metabolism with a characteristic regional pattern of temporoparietal hypometabolism in patients with pathologically confirmed AD [1]. Notable changes in FDG PET scans occur only after subjects become symptomatic. In contrast, in presymptomatic subjects, a relationship between [^11^C]-PIB and [^18^F]-FDG on PET imaging has been previously reported [6]. We have demonstrated that most patients with MCI due to AD based on Aβ and FDG PET biomarkers have a progression to AD, even over the short term [7]. Studies have also reported that CN individuals with markers for both amyloid deposition and neurodegeneration are more likely to develop cognitive impairments during short follow-up periods [8,9]. However, a longitudinal study of Aβ deposition and cerebral glucose metabolism in the subjects with preclinical AD defined by amyloid PET has not been conducted over a long follow-up period.

We defined preclinical AD using amyloid PET imaging for an Aβ biomarker in CN individuals and evaluated the longitudinal change in Aβ deposition and glucose metabolism over the long progression of preclinical AD using PIB PET and FGD PET imaging. In addition, we sought to determine whether Aβ deposition and glucose metabolism are associated with the progression to MCI or AD dementia in the preclinical phase of AD.

## Materials & methods

### Subjects

CN subjects between 60 and 89 years of age were recruited from community-dwelling individuals. The subjects underwent cognitive testing and [^11^C]-PIB PET. The normal cognitive status of CN subjects was defined by a Mini-Mental State Examination (MMSE) score of 28 or more and a clinical dementia rating (CDR) score of 0. Of these subjects, 22 CN subjects who had an amyloid-positive scan were defined as preclinical AD and subsequently included in the long-term follow-up study. Ten CN subjects who had an amyloid-negative scan were also included as the negative CN group. All CN subjects were clinically assessed every 6 months and underwent [^11^C]-PIB PET and [^18^F]-FDG PET imaging at baseline and at least once during the 6.0 ± 1.8 years of follow-up (range 3.1–8.8 years). The apolipoprotein E (APOE) genotype was determined. Participants were excluded if they had a neurodegenerative condition, stroke, psychiatric illness, traumatic brain injury or any other medical condition. Each subject provided written informed consent for participation. The study was approved by the Ethics Committee of the Mirai Iryo Research Center Inc. (Tokyo, Japan).

MCI was diagnosed at each visit if the CN subjects fulfilled the Core Clinical Criteria for MCI proposed by the NIA-AA workgroup [10]. The MMSE score was 24 or more, and the global CDR score was at least 0.5 in the memory domain. Delayed recall of a paragraph from the revised Wechsler memory scale (WMS-R) Logical Memory II (maximum score 25) was used as the measure of the episodic memory. AD dementia was diagnosed based on the criteria of the NIA-AA. AD patients had an MMSE score of 23 or less, a global CDR score of 0.5 or 1 and impaired activities of daily living.

### PET imaging

All PET scans were performed with a Siemens ECAT ACCEL scanner on the same day as cognitive testing. All imaging data were reconstructed into a 128 × 128 matrix with an iterative reconstruction algorithm, using a Gaussian filter with 3.5-mm full-width at half-maximum. The subject's head was immobilized to minimize motion during the scan. Amyloid PET imaging was performed with the radiotracer [^11^C]-PIB [4]. The [^11^C]-PIB was injected with a mean dose of 550.0 ± 10% MBq. Dynamic PET scanning was performed for 60 min using a predetermined protocol. Sixty minutes after the completion of the [^11^C]-PIB PET scan, subjects were injected with 250.0 ± 10% MBq of [^18^F]-FDG and remained in a dark room. Fifteen-minute static FDG PET scans were acquired after a 45-min uptake period.

### Image analysis

A region of interest (ROI) analysis was performed on each individual PET image. All subjects underwent T1-weighted MRI (1.5 T) for coregistration with the PET images. MRI-based correction of the PET data was carried out using PMOD software (PMOD Technologies Ltd, Adliswil, Switzerland). The ROIs were manually drawn on the coregistered MRI of each subject for 20 bilateral cortical regions including the lateral temporal cortex (LTC), medial temporal cortex (MTC), frontal cortex (FC), occipital cortex (OC), parietal cortex (PC), sensory motor cortex (SMC), anterior cingulate gyrus (ACG), posterior cingulate gyrus (PCG), precuneus cortex (Pre) and cerebellar cortex. The cerebellar gray matter was used as a reference region. The ROIs of the follow-up PET images were coregistered with the initial PET images, and the same ROIs were applied to both the baseline and follow-up images.

The retention of [^11^C]-PIB was calculated as the regional-to-cerebellum standardized uptake value ratio (SUVR) for 35–60 min. The regional PIB SUVR in each cortical region and the global cortical PIB SUVR for the mean of the regional SUVR over 18 cortical regions, including LTC, MTC, FC, OC, PC, SMC, ACG, PCG and Pre, were defined. The same coregistration method was applied to the quantification of [^18^F]-FDG. A standardized uptake value of the same regions was normalized to the cerebellar cortex as a reference. Glucose metabolism was referred to as the SUVR, and the global and regional cortical FDG SUVRs were defined. The decrease in glucose metabolism was less than the [^18^F]-FDG SUVR of 0.99 in the cortical regions in our clinical setting, as previously described [7].

The amyloid-positive scan had an increased [^11^C]-PIB SUVR of 1.39 or more in any of the cortical regions at baseline and was classified as a typical or focal positive scan in our clinical setting. The amyloid-negative scan had no [^11^C]-PIB uptake in any cortical region. The cut-point value was based on the bimodal distribution of [^11^C]-PIB uptake in 56 CN controls and 32 AD patients, and it discriminated AD patients from healthy controls with a sensitivity of 97.2% and a specificity of 85.3%, as previously described [11].

### Data management

The subjects underwent clinical assessments and [^11^C]-PIB PET and [^18^F]-FDG PET imaging at three to six time points approximately 12 months apart during the follow-up period. Annual changes in the [^11^C]-PIB and [^18^F]-FDG SUVRs of each cortical region were calculated for each subject at the final follow-up visit using the following equation: annual change = ([SUVR at last follow-up − SUVR at baseline]/follow-up period [year]).

### Statistical analysis

Data were analyzed with Statcel 3 software (OMS, Inc., Tokyo, Japan). Paired *t* tests were used to study changes between baseline and follow-up data. Group differences were evaluated with Bonferroni post hoc tests. Pearson's correlation analyses were conducted for PIB SUVRs, FDG SUVRs, baseline age, MMSE scores, CDR sum of boxes scores and WMS-R recall scores. Categorical variables were examined with Fisher's exact test. The results were considered significant at p < 0.05. Data are presented as the mean ± SD.

## Results

### Clinical data & cognitive function

The demographic characteristics of preclinical AD and amyloid-negative CN subjects at baseline and at follow-up are shown in Table 1. All preclinical AD subjects had a mean age of 71.0 ± 5.9 (range, 62–87) and had no cognitive impairment on MMSE, global CDR or WMS-R Logical Memory II Immediate and Delayed Recall. The proportion of APOE ε4 carriers was greater in preclinical AD subjects (41%) than in CN subjects (10%).

**Table 1.** **Demographic characteristics of preclinical Alzheimer's disease and amyloid-negative cognitively normal subjects at baseline and at follow-up.**

	**Preclinical Alzheimer's disease**			**Cognitively normal**

	**All**	**Converter**	**Stable**	
n	22	12	10	10

Female	9 (41%)	5 (41%)	4 (40%)	5 (50%)

Age (y)	71.0 ± 5.9	71.1 ± 5.8	70.9 ± 6.1	65.7 ± 3.4

Education (y)	12.1 ± 2.6	12.2 ± 2.4	11.9 ± 2.7	13.5 ± 1.6

APOE ε4 carriers	9 (41%)	4 (33%)	5 (50%)	1 (10%)

At baseline				

MMSE	29.1 ± 0.8	29.0 ± 0.7	29.2 ± 0.8	29.6 ± 0.6

Global CDR	0	0	0	0

Immediate Rec	13.3 ± 2.5	12.7 ± 2.6	14.1 ± 2.1	14.2 ± 1.8

Delayed Rec	11.6 ± 2.7	10.6 ± 3.0	12.9 ± 1.2	12.1 ± 3.0

At the last follow-up				

MMSE	27.1 ± 2.4	25.7 ± 2.5^†^	28.8 ± 0.7	29.0 ± 0.6

Global CDR	0.25 ± 0.25	0.5	0	0

Immediate Rec	9.9 ± 4.6	7.1 ± 3.8^†^	13.2 ± 3.2	13.8 ± 1.9

Delayed Rec	7.7 ± 4.5	4.6 ± 3.4^†^	11.4 ± 2.7	12.4 ± 3.4

Follow-up duration (y)	6.0 ± 1.8	6.5 ± 1.3	5.4 ± 2.3	5.6 ± 2.6

Data are presented as means ± SD.

^†^Statistically significant difference from baseline and CN by two-sample t-tests (p < 0.05).

APOE: Apolipoprotein E; CDR: Clinical dementia rating; CDR SB: CDR sum of boxes; MMSE: Mini-Mental State Examination; n: Number of patients; Rec: WMS-R recall scores; WMS-R: Revised Wechsler memory scale; y: Year.

**Figure 1.**
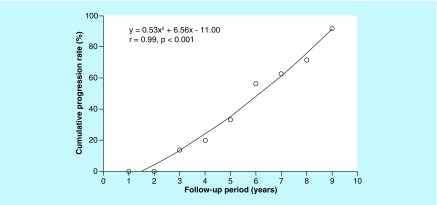
**Curvilinear regression between the consecutive rate of progression from preclinical Alzheimer's disease to mild cognitive impairment and the follow-up period in preclinical Alzheimer's disease subjects.** The linear model is y = -11.63 + 7.19x + 0.46x^2^ (r = 0.99; p < 0.001).

12 (55%) of the 22 subjects with preclinical AD had a progression to MCI during the mean follow-up of 6.0 ± 1.8 years (converters), whereas the other ten subjects remained clinically stable (stable). The pattern of the consecutive progression rate of preclinical AD to MCI best fit a curvilinear regression line (Figure 1). Three (14%) of these 22 subjects progressed to MCI within 3 years after baseline, and ten (63%) of 16 subjects progressed within 7 years. The overall rate of preclinical AD progression to MCI was 10.4% per year. Two of the preclinical AD subjects developed AD dementia during the follow-up period, but none of the amyloid-negative CN subjects progressed to MCI or any dementia.

When preclinical AD subjects progressed to MCI, the mean MMSE score significantly decreased from 29.0 ± 0.7 to 25.7 ± 2.5 (n = 12; p < 0.05) and the global CDR score was 0.5. The mean delayed paragraph recall score of converters was 4.6 ± 3.4 (n = 12; p < 0.05), which was significantly different from that of stable subjects despite their similar level of education.

Nine (56%) of 16 preclinical AD subjects aged 65–75 years progressed to MCI. Two (66.6%) of three preclinical AD subjects younger than age 64 years progressed to MCI, whereas one (33.3%) of three subjects older than age 76 years progressed. There was no significant difference in the progression rate of three age subgroups (*χ* ^2^ p = 0.74). Five (55%) of nine women with preclinical AD progressed to MCI, which was not significantly different from seven (53%) of 13 men (Fisher's exact p = 0.63). Four (44.4%) of nine APOE ε4 carriers with preclinical AD progressed to MCI, whereas eight (62%) of 13 APOE ε4 noncarriers progressed. There was no significant difference in the progression rate between APOE ε4 carriers and noncarriers (Fisher's exact p = 0.36). The progression to MCI was not directly related to baseline age, gender or APOE ε4 status.

### Aβ deposition

18 of 22 preclinical AD subjects had marked [^11^C]-PIB uptake in the FC, PC and LTC regions as well as in the cingulate gyrus and precuneus on the typical positive scan. In contrast, four subjects had focal positive scans with increased regional [^11^C]-PIB uptake in the Pre, PC and/or FC regions.

The mean value of global cortical PIB SUVRs in preclinical AD subjects at baseline (1.60 ± 0.23; n = 22; p < 0.01) was significantly higher than that in amyloid-negative CN subjects (1.15 ± 0.04; n = 10). The mean of the global cortical PIB SUVR at follow-up (1.89 ± 0.28; n = 22; p < 0.01) was significantly increased more than that at baseline. The annual increase in the global cortical SUVR in preclinical AD subjects over a mean follow-up of 6.0 ± 1.8 years was 0.054 ± 0.020 (n = 22; p < 0.01), which was significantly larger than that in amyloid-negative CN subjects (0.005 ± 0.007; n = 10). This change was a 3.3% annual increase in the PIB SUVR from baseline.

The global cortical PIB SUVR in APOE ε4 carriers with preclinical AD at baseline (1.59 ± 0.28; n = 9; p = 0.96) did not significantly differ from that in APOE ε4 noncarriers (1.60 ± 0.21; n = 13). There was also no significant difference in the annual change in PIB SUVR between APOE ε4 carries and noncarriers. In addition, the baseline age of individual preclinical AD subjects was not significantly related to the baseline PIB SUVR (r = -0.23; n = 22; p = 0.29) or the annual change in the PIB SUVR (r = 0.14; n = 22; p = 0.52).

**Figure 2.**
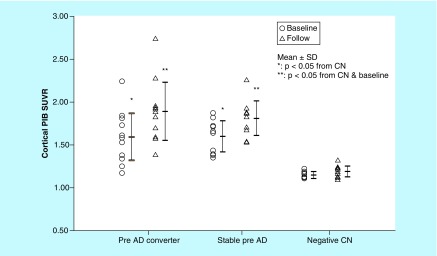
**Cortical Pittsburgh Compound-B standardized uptake value ratio in preclinical Alzheimer's disease (pre-Alzheimer's disease) converters (n = 12), stable preclinical Alzheimer's disease subjects (n = 10) and amyloid-negative cognitively normal subjects (n = 10) at baseline (open circles) and follow-up (open triangles).** Data are presented as the mean ± SD. *Statistically significant compared with CN (p < 0.05); **Statistically significant compared with baseline and negative CN (p < 0.05). CN: Cognitively normal; PIB: ^11^C-labelled Pittsburgh Compound-B; SD: Standard deviation; SUVR: Standardized uptake value ratio.

**Figure 3.**
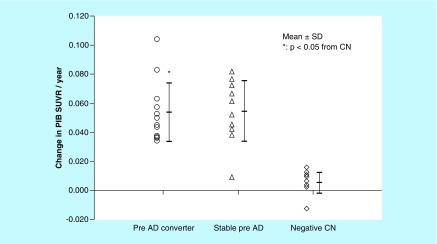
**Annual changes in the cortical Pittsburgh Compound-B standardized uptake value ratio of preclinical Alzheimer's disease (pre-Alzheimer's disease) converters (n = 12), stable preclinical Alzheimer's disease subjects (n = 10) and amyloid-negative cognitively normal subjects (n = 10).** The annual increase in the cortical PIB SUVR in preclinical AD converters and stable subjects is significantly greater than in negative CN subjects. Data are presented as the mean ± SD. *Statistically significant compared with negative CN (p < 0.05). AD: Alzheimer's disease; CN: Cognitively normal; PIB SUVR: Pittsburgh Compound-B standardized uptake value ratio; SD: Standard deviation.

When preclinical AD subjects were separated into two subgroups of converters and stable subjects, the mean cortical PIB SUVR at baseline in preclinical AD converters (1.59 ± 0.27; n = 12; p = 0.99) was similar to that in stable subjects (1.60 ± 0.18; n = 10; Figure 2). The annual increase in the global cortical SUVR in preclinical AD converters was 0.053 ± 0.020 (n = 12; p = 0.91), which was similar to 0.054 ± 0.021 (n = 10) in stable subjects (Figure 3). In contrast, the period of progression to MCI in individual preclinical AD converters was significantly related to the cortical PIB SUVR at baseline (r = -0.57; n = 12; p < 0.05) but not the annual increase in the cortical PIB SUVR (r = -0.52; n = 12; p = 0.08).

Among the cortical regions of preclinical AD subjects, the regional PIB SUVRs at baseline and the annual increases in the regional cortical PIB SUVR in all cortical regions except MTC were significantly higher than those in the amyloid-negative CN subjects (Table 2). The regional PIB SUVRs at baseline (1.89 ± 0.39; n = 22) and the annual increase in the regional PIB SUVR (0.080 ± 0.036; n = 22) were greatest in the Pre. There was no significant difference in the regional baseline PIB SUVR and the annual change in the PIB SUVR of any cortical region between converters and stable subjects.

**Table 2.** **Regional Pittsburgh Compound-B standardized uptake value ratio at baseline and the annual change of regional Pittsburgh Compound-B standardized uptake value ratio in preclinical Alzheimer's disease subjects and negative-amyloid cognitively normal subjects.**

	**Baseline**		**Annual change**	

	**Pre-Alzheimer's disease**	**Cognitively normal**	**Pre-Alzheimer's disease**	**Cognitively normal**
n	22	10	22	10

Medial temporal cortex	1.18 ± 0.12	1.06 ± 0.12	0.005 ± 0.027	-0.001 ± 0.022

Lateral temporal cortex	1.60 ± 0.27^†^	1.16 ± 0.10	0.063 ± 0.029^†^	0.004 ± 0.012

Anterior cingulate gyrus	1.76 ± 0.42^†^	1.19 ± 0.10	0.068 ± 0.041^†^	-0.002 ± 0.023

Frontal cortex	1.78 ± 0.36^†^	1.16 ± 0.11	0.051 ± 0.032^†^	0.006 ± 0.0184

Occipital cortex	1.37 ± 0.18^†^	1.21 ± 0.08	0.047 ± 0.033^†^	-0.009 ± 0.009

Posterior cingulate gyrus	1.71 ± 0.35^†^	1.22 ± 0.12	0.063 ± 0.035^†^	0.011 ± 0.015

Precuneus	1.89 ± 0.39^†^	1.15 ± 0.07	0.080 ± 0.036^†^	0.011 ± 0.0162

Parietal cortex	1.70 ± 0.36^†^	1.09 ± 0.11	0.060 ± 0.038^†^	0.002 ± 0.017

Sensory motor cortex	1.45 ± 0.28^†^	1.17 ± 0.10	0.028 ± 0.043^†^	-0.002 ± 0.015

Data are presented as mean ± standard deviation.

^†^Statistically significant difference from baseline by two-sample t-tests (p < 0.05).

### Glucose metabolism

**Figure 4.**
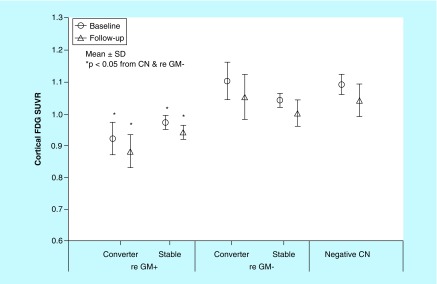
**Cortical fluorodeoxyglucose standardized uptake value ratio in preclinical Alzheimer's disease converters and stable subjects with reduced glucose metabolism (re-GM^+^; n = 11) and without reduced glucose metabolism (re-GM^-^, n = 11), and amyloid-negative cognitively normal subjects (n = 10) at baseline (open circles) and follow-up (open triangles).** Data are presented as the mean ± SD. *Statistically significant difference with multiple comparisons post hoc tests (p < 0.05).

None of the ten CN subjects with negative-amyloid scans had reduced glucose metabolism in any cortical region over the follow-up period. The mean global cortical FDG SUVR at baseline and follow-up were 1.07 ± 0.02 (n = 10) and 1.05 ± 0.04 (n = 10), respectively (Figure 4).

11 (50%) of 22 subjects with preclinical AD had reduced glucose metabolism in AD-affected regions, such as the LTC, PC and/or Pre. The mean global cortical FDG SUVR decreased from 0.94 ± 0.05 at baseline to 0.91 ± 0.05 (n = 11, p < 0.01) at follow-up (Figure 4). The mean annual change in the cortical FDG SUVR was -0.006 ± 0.006 (n = 11). In contrast, the 11 subjects without reduced glucose metabolism had a global FDG SUVR of 1.07 ± 0.05 at baseline, which did not change at follow-up. Six (54%) of 11 preclinical AD subjects with reduced glucose metabolism progressed to MCI, which was not significantly different from the six (54%) of 11 subjects without reduced glucose metabolism (Fisher's exact p = 0.66). In preclinical AD subjects with reduced glucose metabolism, there was no significant difference in the baseline FDG SUVR or the annual change in FDG SUVR between converters and stable subjects.

Of the AD-affected cortical regions, seven (58%) of 12 subjects with reduced glucose metabolism (FDG SUVR: 0.94 ± 0.04; n = 12) in the LTC progressed to MCI, whereas five (50%) of ten subjects without reduced glucose metabolism progressed. In addition, three (43%) of seven subjects with reduced glucose metabolism (FDG SUVR: 0.93 ± 0.04; n = 7) in the PC progressed to MCI, whereas nine (60%) of 15 subjects without reduced glucose metabolism progressed. There was no significant difference in the rate of MCI progression between subjects with and without reduced glucose metabolism in the LTC (Fisher's exact p = 0.51) or in the PC (Fisher's exact p = 0.38). The reduced glucose metabolism in AD-affected regions of preclinical AD subjects was not related to the progression to MCI.

### Aβ deposition & glucose metabolism

**Figure 5.**
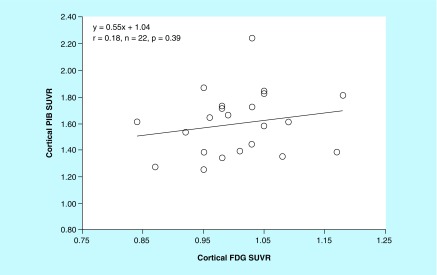
**Relationship between cortical Pittsburgh Compound-B and fluorodeoxyglucose standardized uptake value ratio values at baseline in individual preclinical Alzheimer's disease subjects.** Cortical PIB SUVRs are not significantly correlated with cortical FDG SUVRs (r = 0.18, n = 22; p = 0.39). FDG SUVR: Fluorodeoxyglucose standardized uptake value ratio; PIB SUVR: Pittsburgh Compound-B standardized uptake value ratio.

**Figure 6.**
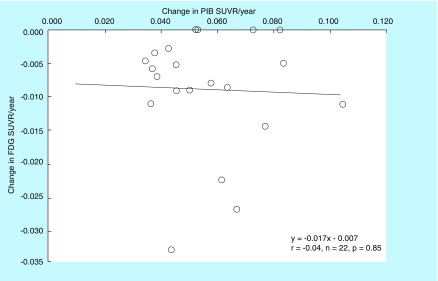
**Relationship between annual changes in the cortical Pittsburgh Compound-B standardized uptake value ratio and fluorodeoxyglucose standardized uptake value ratio in individual preclinical Alzheimer's disease subjects.** The annual change in the PIB SUVR is not significantly correlated with that in the FDG SUVR (r = -0.04; n = 22; p = 0.85). FDG SUVR: Fluorodeoxyglucose standardized uptake value ratio; PIB SUVR: Pittsburgh Compound-B standardized uptake value ratio.

The relationship between the global cortical PIB SUVR and the FDG SUVR in individual preclinical AD subjects at baseline is shown in Figure 5. Individual cortical PIB SUVRs were not significantly correlated with cortical FDG SUVRs in preclinical AD subjects (r = 0.21; n = 22; p = 0.33). Annual changes in the cortical PIB SUVR in individual preclinical AD subjects were not significantly correlated with those in the cortical FDG SUVR (r = -0.04; n = 22; p = 0.85; Figure 6). Among AD-affected cortical regions, there were no significant differences in the baseline value or the annual change between the PIB SUVR and the FDG SUVR in the PC or LTC.

In preclinical AD converters, the individual cortical PIB SUVRs at baseline were not significantly correlated with cortical FDG SUVRs (r = 0.32; n = 12; p = 0.30). In addition, the individual annual change in the cortical PIB SUVR was not correlated to that in the cortical FDG SUVR (r = 0.04; n = 12; p = 0.96).

## Discussion

We demonstrated that 12 (55%) of 22 subjects with preclinical AD progressed to MCI during 6.0 ± 1.8 years of long-term follow-up. The age, gender and APOE ε4 status of the subjects were not associated with the progression to MCI. A previous study described that the rate of progression to MCI was 18% over a short follow-up of 15 months in 90 cognitively normal participants with amyloid positivity (preclinical AD stages 1–3) [8]. Additionally, the Australian Imaging, Biomarkers and Lifestyle research group reported that eight (25%) of 32 healthy individuals with positive Aβ PET scans progressed to MCI by 3 years [12]. Our findings indicate that a higher proportion of the CN subjects with Aβ deposition have progression to MCI if we have much longer observation periods. It is suggested that individuals with preclinical AD progress to MCI over the long term when preclinical AD is defined by positive PET imaging of Aβ biomarkers, although some older individuals with preclinical AD may not become symptomatic during their lifetime. Furthermore, the present study showed that the overall rate of preclinical AD progression to MCI was 10.4% per year. This rate is lower than the 23.4% per year of MCI due to AD progression to AD that was previously reported [5]. Subjects with preclinical AD cloud progress more slowly than subjects with MCI due to AD.

When subjects with preclinical AD had reduced glucose metabolism with FDG PET imaging at baseline, the present study found that six (54%) of 11 subjects with reduced glucose metabolism progressed to MCI over a long follow-up of 6.0 ± 1.8 years. This rate of progression did not significantly differ from the six (54%) of 11 subjects without reduced glucose metabolism. A previous study reported that the rate of conversion to MCI in 25 individuals with preclinical AD stages 2 + 3 with β-amyloidosis and lower glucose metabolism was 21% over a short follow-up of 15 months, which was not different from the 19% of 32 individuals with preclinical AD stage 1 with β-amyloidosis alone [13]. We suggest, in subjects with preclinical AD, that a reduction in cerebral glucose metabolism at baseline could not be predict of a progression to MCI even over a longer time.

We demonstrated that the global cortical PIB SUVR in preclinical AD subjects at follow-up significantly increased from baseline. The annual increase in the PIB SUVR was 0.054 ± 0.020 and 3.3% from baseline. This is consistent with a longitudinal PIB PET study that PIB SUVR was 0.046 ± 0.03 per year in healthy controls with PIB retention [14]. These findings indicate that Aβ deposition continues to accumulate over time in subjects with preclinical AD. However, the present study found that the annual increase in Aβ deposition in the preclinical AD converters did not significantly differ from that that in stable subjects during the follow-up period. On the other hand, the cortical PIB SUVR at baseline in preclinical AD converters was correlated with the period of progression to MCI. These findings suggest that subjects with preclinical AD can progress to MCI even over a shorter period when they have higher Aβ deposition at baseline in addition to a continuous increase in Aβ deposition.

We found that 11 (50%) of 22 preclinical AD subjects had reduced cerebral glucose metabolism at baseline in AD-affected regions of the LTC, PC and/or Pre. The mean cortical FDG SUVR significantly decreased from 0.94 ± 0.05 at baseline to 0.91 ± 0.05 during a long follow-up of 6 years, and the annual change in the FDG SUVR was -0.0061 ± 0.0060. This result is consistent with a previous study that the hypometabolism in the AD signature composite regions at baseline decreased over a short follow-up of 15 months in 43% of CN participants with amyloid deposition [15]. In addition, the present study showed, in preclinical AD subjects with reduced glucose metabolism, that there was no significant difference in the baseline FDG SUVR or the annual change in the FDG SUVR between converters and stable subjects. These findings indicate that a change in glucose metabolism is not associated with the progression to MCI. We suggest that if preclinical AD subjects have reduced glucose metabolism in AD-affected regions at baseline the glucose metabolism could further decrease over time without being directly involved in the progression to MCI.

The present study demonstrated that the cortical PIB SUVRs at baseline were not significantly correlated with cortical FDG SUVRs in individual preclinical AD subjects. Furthermore, the annual increase in Aβ deposition was not significantly related to the annual decrease in glucose metabolism. These findings support the previous study that the abnormal levels of Aβ did not result in greater hypometabolism in CN individuals [6]. In AD patients, in contrast, higher Aβ retention has already established to be correlated with lower glucose metabolism in the temporal and parietal cortices. Early in the preclinical stage of AD spectrum, the volume and period of increased Aβ deposition may be not sufficient to directly promote hypometabolism. It appears that there are different upstream causes for Aβ production and glucose metabolism.

We demonstrated that subjects with preclinical AD more frequently progressed to MCI over a long follow-up period when preclinical AD was defined by a positive PIB PET scan of the Aβ biomarker. In addition, our study showed that Aβ deposition continuously increased over time in preclinical AD subjects, but the increase in Aβ deposition was not directly related to decrease in glucose metabolism even in AD-affected cortical regions. The Aβ deposition may be the primary event that induces other neurodegenerative processes, such as the accumulation and/or spreading of Tau, in the early stage of preclinical AD. Therefore, if preclinical AD is defined by amyloid PET imaging, a great success may be achieved with anti-amyloid agents by decreasing the production and accumulation of Aβ very early in the disease process, before clinical impairment.

Certain limitations of our study should be noted. We conducted a successful longitudinal assessment of Aβ deposition and glucose metabolism on the progression of preclinical AD subjects during a long-term follow-up period, but the number of preclinical AD subjects was small. The study population may have been limited by the definition of CN and the threshold for defining amyloid positivity and reduced glucose metabolism in our clinical setting.

## Conclusion

Preclinical AD subjects have continuous increases in Aβ deposition over time independent of glucose metabolism. Increased Aβ deposition leads to a progression to MCI but reduced glucose metabolism does not contribute to the progression. Subjects with preclinical AD defined by amyloid PET are at a higher risk for progression to MCI or AD dementia over the long term.

## Future perspective

Cognitively normal subjects progress to MCI or AD over a long period if they have Aβ deposition as evaluated by a positive amyloid PET scan. The Aβ deposition continuously increases over time and induces other neurodegenerative processes. Antiamyloid agents that decrease the production and/or accumulation of Aβ should be developed in the future, and are needed very early in the disease process, before clinical impairment.

**Summary points**

- Subjects with preclinical Alzheimer's disease (AD) progress to mild cognitive impairment (MCI) over the long term if preclinical AD is defined by amyloid positron emission tomography imaging.
- The cortical Pittsburgh Compound-B standardized uptake value ratio (PIB SUVR) is higher at baseline and increases during follow-up period in preclinical AD.
- The annual increase in the PIB SUVR is 3.3% from baseline.
- In preclinical AD subjects who progressed to MCI, the baseline value or the annual change in cortical PIB SUVR is not significantly correlated with that in cortical fluorodeoxyglucose SUVR.
- A reduction in cerebral glucose metabolism is not correlated with a progression to MCI even over a longer time.
- Increases in amyloid β deposition lead to the progression to MCI but decreases in glucose metabolism do not contribute to progression in the preclinical stage of AD spectrum.
